# Successful treatment of adult‐onset nesidioblastosis by continuous subcutaneous octreotide infusion in a patient on hemodialysis

**DOI:** 10.1002/ccr3.3514

**Published:** 2020-11-11

**Authors:** Rina Kato, Akihiro Nishimura, Kimio Matsumura, Shota Kikuno, Kaoru Nagasawa, Yasumichi Mori

**Affiliations:** ^1^ Department of Endocrinology and Metabolism Toranomon Hospital Minato‐ku Japan

**Keywords:** hemodialysis, nesidioblastosis, octreotide

## Abstract

Octreotide may be useful in noninsulinoma pancreatogenous hypoglycemia syndrome with nesidioblastosis patients who was on hemodialysis. Continuous octreotide subcutaneous infusion can reduce side‐effects and stabilize plasma glucose levels.

## Graphical abstract

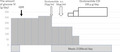

## 1 INTRODUCTION

We reported a rare case of adult‐onset noninsulinoma pancreatogenous hypoglycemia syndrome with nesidioblastosis on regular hemodialysis that was successfully treated with pancreaticoduodenectomy and octreotide. Continuous subcutaneous infusion of octreotide may be an effective treatment for patients on hemodialysis and cannot tolerate bolus subcutaneous injection of octreotide.

Noninsulinoma pancreatogenous hypoglycemia syndrome (NIPHS) with Nesidioblastosis was first described by Laidlaw in 1938^1^ as a condition characterized by persistent hyperinsulinemic hypoglycemia (PHH) associated with diffuse neoformation of Langerhans islet β cells. It is the main cause of PHH in infants, though it is extremely rare in adults. The diagnosis requires proof of hyperinsulinemia and pathological features. It is difficult to distinguish NIPHS with nesidioblastosis from insulinoma before tumor sampling. Since the size of insulinoma is often <2 cm, localization diagnosis is difficult by images. Arterial stimulation venous sampling (ASVS) is useful for localization and recognition of insulinoma. When the surgical resection of the pancreas for the treatment of NIPHS with nesidioblastosis fails or is considered to be too risky, several medications (diazoxide, octreotide, and corticosteroids) are used.^2^ Here, we report an adult‐onset NIPHS with nesidioblastosis case that showed uncontrolled hypoglycemia after pancreaticoduodenectomy and was treated successfully with continuous subcutaneous infusion (CSI) of octreotide.

## 2 CASE REPORT

A 73‐year‐old man was admitted to our hospital because he had repeated episodes of hypoglycemia before meals for 4 months. He suffered from chronic glomerulonephritis and had been on dialysis for 18 years.

**FIGURE 1:**
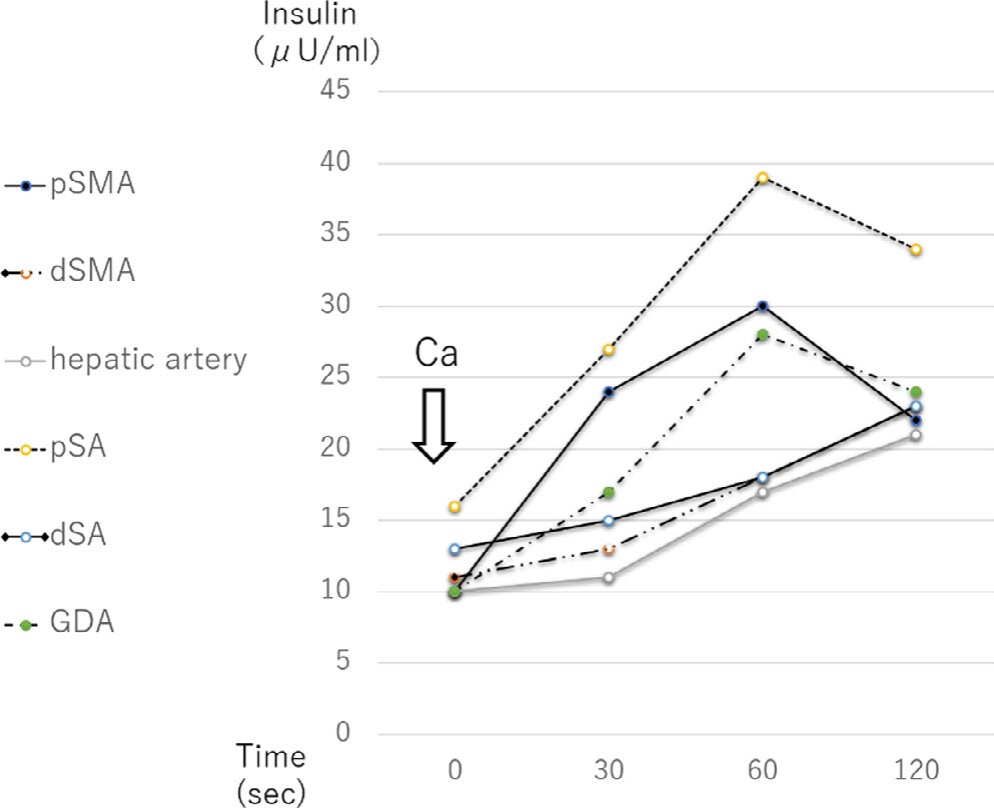
Results of Selective arterial calcium stimulation test. Ca, calcium stimulation; dSA, distal splenic artery; dSMA, distal superior mesenteric artery; GDA, gastroduodenal artery; PHA, proper hepatic artery; pSA, proximal splenic artery; pSMA, proximal superior mesenteric artery

Body height and weight were 159 cm and 65.1 kg, respectively, and physical examination was almost normal. Analysis of the daily energy intake pattern showed meals of 1800 kcal/d and snacks of 200 kcal thrice daily, although the plasma glucose level was 40‐70 mg/dL throughout the day. Glycemic support was provided in the form of continuous infusion of 245 g/24 h (10 g/h) dextrose. The results of laboratory tests showed hypoglycemia, hyperinsulinemia, high C‐peptide, low HbA1c, and mild hypocarnitinemia (Table 1). He was treated with carnitine 1000 mg intravenously after each hemodialysis, but this did not improve hypoglycemia. Enhanced abdominal CT identified a 5 mm‐hypervascular tumor in the duodenal wall. All images were negative for pancreatic lesions. The results of ASVS were interesting; injection of calcium gluconate resulted in increase in serum insulin levels in the proximal superior mesenteric artery (pSMA), proximal splenic artery (pSA), gastroduodenal artery (GDA) within 60 seconds, together with increase within 120 seconds in all other regions (Figure 1). Since the case fulfilled the Whipple's triad and positive findings of ASVS were detected in pancreatic head arteries, the provisional diagnosis was a small pancreatic head insulinoma that could not be detected by images.

**Table 1:** Laboratory data on admission

WBC	8.1 × 10^3^/µL	TP	6.7 g/dL
RBC	4.2 × 10⁶/µL	Alb	3.3 g/dL
Hb	10.9 g/dL	AST	12 IU/L
Plt	21.5 × 10^3^/µL	ALT	14 IU/L
GH	0.9 ng/mL	LDH	197 IU/L
IGF‐1	162 ng/mL	ALP	198 IU/L
ACTH	59.9 pg/mL	γ‐GT	21 IU/L
Cortisol	13.5 µg/dL	T‐Bil	1.6 mg/dL
Adrenaline	0.11 ng/mL	Glucose	46 mg/dL
Noradrenaline	0.5 ng/mL	HbA1c	4.7%
Dopamine	0.01 ng/mL	BUN	38 mg/dL
IRI	18 µU/mL	Cr	10.09 mg/dL
C‐peptide	9.17 ng/mL	UA	6.0 mg/dL
Glucagon	349 pg/mL	Na	139 mmol/L
Insulin antibody	<0.4 IU/mL	K	4.2 mmol/L
TSH	3.68 µIU/mL	Cl	103 mmol/L
F‐T3	3.51 pg/mL	Ca	9.0 mmol/L
F‐T4	0.92 ng/dL	P	3.4 mmol/L
TSH receptor Ab	<0.3 IU/L	CRP	0.1 mg/dL
Intact PTH	326 pg/mL		
Total Carnitine	28.9 µmol/L	(45.0‐91.0)	
Free Carnitine	17.6 µmol/L	(36.0‐74.0)	
Acylcarnitine	11.3 µmol/L	(6.0‐23.0)	

Abbreviations: ACTH, adenocorticotropic hormone; F‐T3, free triiodothyronine; F‐T4, free thyroxine; GH, growth hormone; IGF‐1, insulin‐like growth factor‐1; PTH, parathyroid hormone; TSH, thyroid‐stimulating hormone.

The gastroenterology surgery team performed tumor sampling. Although intraoperative ultrasound detected a hypoechogenic lesion in the pancreatic head, histopathological examination of the obtained biopsy material was negative for malignancy. The duodenal submucosal tumor and 13a lymph nodes were also negative for discrete lesions. No tumor could be detected during the operation, and accordingly, pancreaticoduodenectomy was performed with resection of approximately 70% of the pancreas.

**FIGURE 2:**
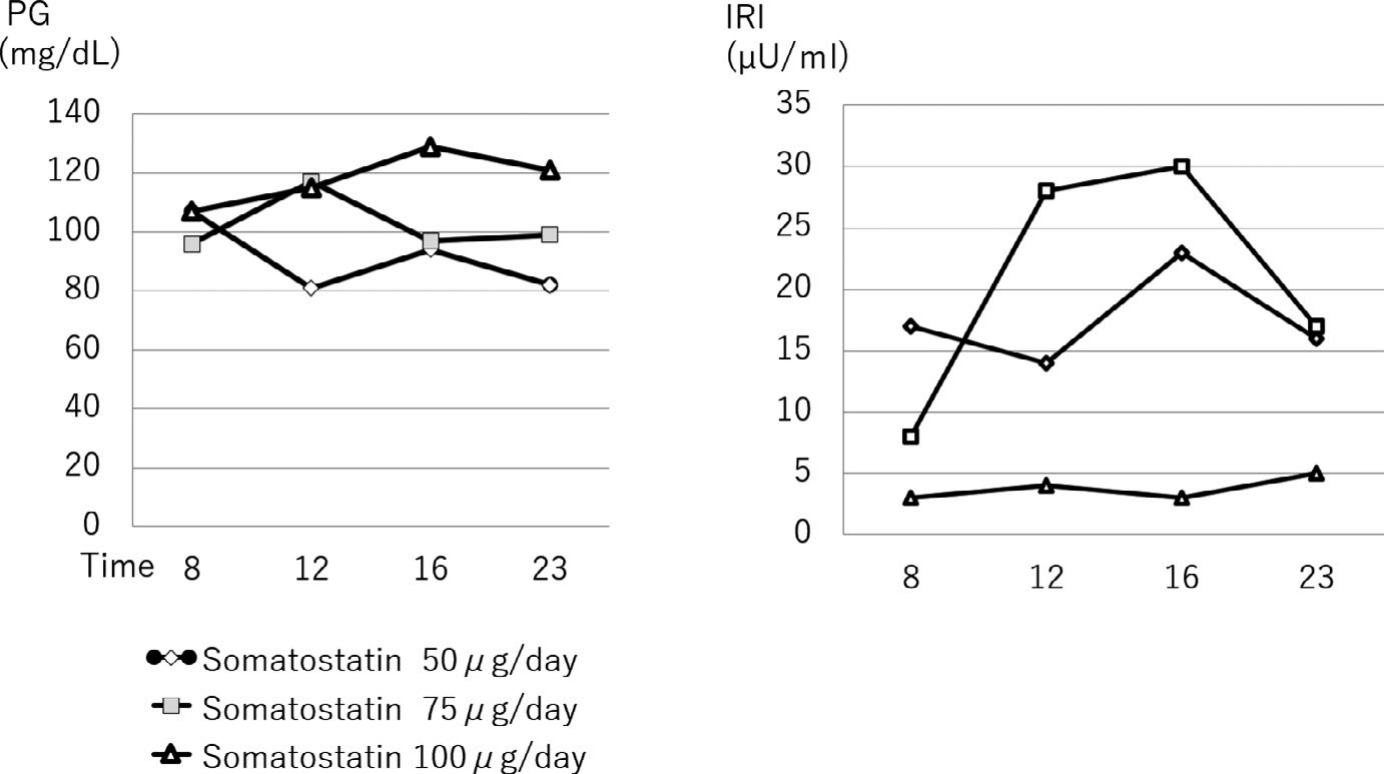
Daily profile of plasma glucose (mg/dL) and insulin (µU/mL) after administration of octreotide at 50 µg/d (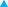), 75 µg/d ((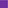), and 100 µg/d ((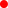)

**FIGURE 3:**
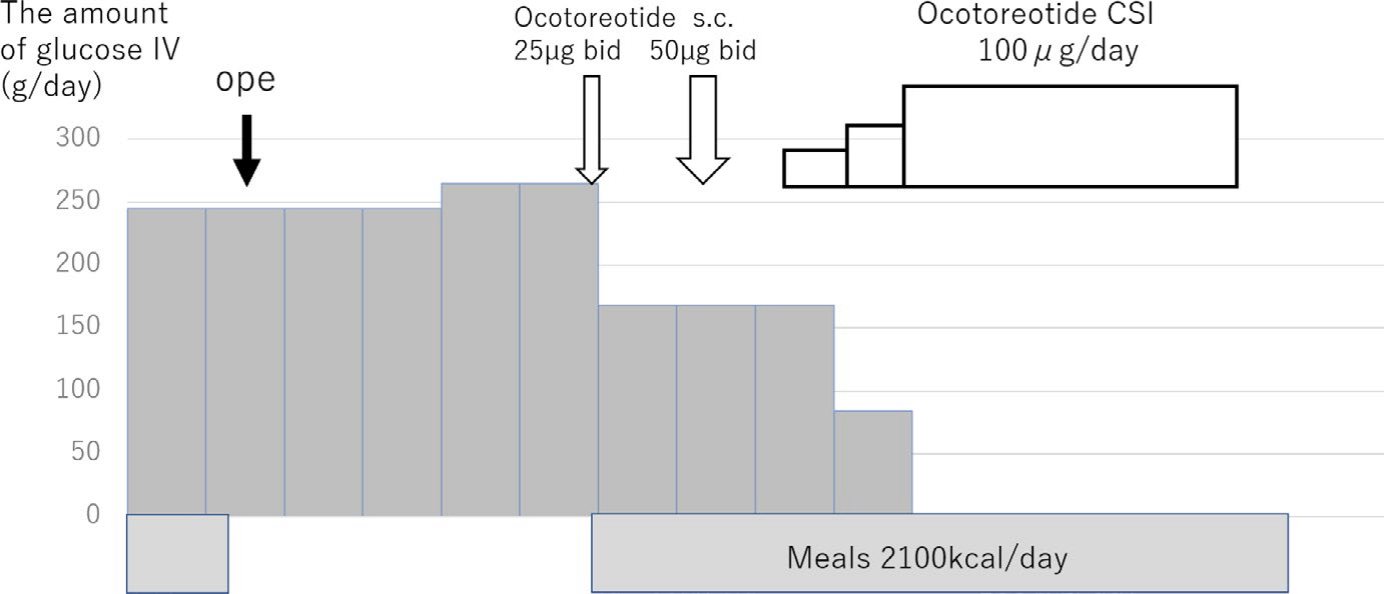
Clinical course of the patient. 20 d after surgery, he was still dependent on glycemic support despite the standard meals. bid, twice a day; CSI, continuous subcutaneous injection; IV, intravenous; s.c, subcutaneous injection

Surgery did not alleviate PHH and the patient continued to require glycemic support. The histopathological findings were consistent with the diagnosis of nesidioblastosis. The patient was treated with a hormone suppressing regimen using octreotide. Octreotide was effective against hyperinsulinemia as confirmed by the results of octreotide loading test, although the dose induced severe nausea and vomiting. We started bolus subcutaneous injection (BSI) of 25 µg octreotide twice a day, but the dose failed to suppress insulin level. On the other hand, 50 µg twice a day induced side‐effects. In the next step, we replaced BSI with CSI, which resulted in the disappearance of the side‐effects, and we were able to increase the dose up to 100 µg/24 h. This regimen resulted in clinical meaningful insulin suppression and glucose level control (Figure 2). Attempts to increase the dose to 150 µg/24 h to improve nighttime hypoglycemia failed due to the appearance of side‐effects. Accordingly, prednisolone at 5 mg/d at evening was added. The combination therapy prevented hypoglycemia (Figure 3). Two years after discharge, the patient showed marked improvement in quality of life while on the same combination treatment.

## 3 DISCUSSION

We experienced a case of adult‐onset NIPHS with nesidioblastosis with uncontrolled hypoglycemia after pancreaticoduodenectomy. It was difficult to explain the result of ASVS and to select the most suitable treatment especially in our patient who was on hemodialysis.

Determination of the cause of hypoglycemia in patients with chronic kidney disease (CKD) requires careful attention. In CKD patients, glucose intolerance due to peripheral resistance to insulin is common and these patients sometimes present with mild to moderate hyperinsulinemia.^3^ Impaired renal degradation of insulin is associated with gluconeogenesis or reduced caloric intake, thus enhancing the development of hypoglycemia. In addition, attention should be paid to carnitine deficiency especially in hemodialysis patients. Insulinoma or nesidioblastosis was suspected in our patient based on the persistent hypoglycemia and fulfillment of the Whipple's triad after normalization of serum carnitine level. The results of ASVS were somewhat confusing based on the different between 60 and 120 seconds. Based on the histopathological diagnosis of nesidioblastosis, we considered renal degradation of insulin could affect the results. Serum insulin levels before stimulation is considered to be higher in CKD patients than non‐CKD patients due to renal degradation of insulin, so that they may be more difficult to meet the criteria. If the above was true, one can explain the results of ASVS based on the diagnosis of nesidioblastosis. The mean maximum and mean relative‐fold increase in insulin may help establish the diagnosis. One previous study reported significantly higher mean maximum level and relative‐fold increase insulin of ASVS in patients with insulinoma than those with nesidioblastosis.^4^ Both results in our case were similar to those of nesidioblastosis.

It is known that partial pancreatectomy of 60%‐80% controls NIPHS with nesidioblastosis in 50% of patients without additional medications.^5^ Unfortunately, pancreaticoduodenectomy failed to control the disease in our case, but we did not recommend subtotal pancreatectomy, due to the known risk of development of insulin‐dependent diabetes mellitus in 40% of patients.^5^ Diazoxide is the most frequently used agent in infant and adult PHH, but fluid retention is the most common side‐effect. There are only three previously reported cases of use of diazoxide in hemodialysis patients, and one of these patients died with heart failure after the administration.^6^, ^7^, ^8^ On the other hand, octreotide, a somatostatin analogue, has been used for PHH in adult patients with inadequate response or resistant to diazoxide. CSI of Octreotide is one of the second‐line therapies for pediatric patients with congenital hyperinsulinism,^9^ although it is not approved in Japan for this indication. Especially, there are few reports about adult‐onset NIPHS with nesidioblastosis treated with CSI of octreotide. It was for these reasons we chose octreotide rather than diazoxide in our case that octreotide is metabolized predominantly in the liver and has been often used in hemodialysis patients.^10^

We also showed that the use of octreotide in CSI avoided gastrointestinal symptoms compared with BSI. While the actual mechanism is still unknown, we believe the symptoms are related to peak plasma concentrations. In fact, the peak concentration at 100 µg octreotide observed during BSI was higher than 300 µg/24 h with CSI.^11^ CSI does not only reduce peak plasma concentrations, avoid side‐effects, and allow increase in total dosage but also reduce times of injection and tachyphylaxis.^12^ Further studies are needed to determine whether long‐acting octreotide (Sandostatin‐LAR^®^) is better or not. In this regard, Kondo et al^13^ reported a case treated with Sandostatin‐LAR for a Japanese adult‐onset NIPHS with nesidioblastosis. Sandostatin‐LAR can only be administered in our case after testing its safety and efficacy in hemodialysis patients.

In conclusion, we reported a case of adult‐onset NIPHS with nesidioblastosis on regular hemodialysis that was successfully treated with pancreaticoduodenectomy and octreotide. CSI of octreotide may be an effective treatment for patients who cannot tolerate BSI.

## CONFLICT OF INTEREST

None of the authors have any potential conflicts of interest with this report.

## AUTHOR CONTRIBUTIONS

RK and AN: involved in writing and editing the manuscript. KM, SK, KN, and YM: involved in revision of the manuscript. All authors: involved in reading and approving final version of the manuscript.

## ETHICS APPROVAL AND CONSENT TO PARTICIPATE

This case report was reviewed and approved by the ethics committee of Toranomon Hospital with the identifier No. 1851.

## Data Availability

The datasets generated and analyzed during the current study available from the corresponding author on reasonable request.

## References

[1] Laidlaw GF . Nesidioblastoma, the islet tumor of the pancreas. Am J Pathol. 1938;14:125‐134. 19970380 PMC1964945

[2] Dravecka I , Lazurova I . Nesidioblastosis in adults. Minireview. Neoplasma. 2014;61:252‐256. 24645840 10.4149/neo_2014_047

[3] Abe M , Kaizu K , Matsumoto K . Plasma insulin is removed by hemodialysis: evaluation of the relation between plasma insulin and glucose by using a dialysate with or without glucose. Ther Apher Dial. 2007;11:280‐287. 17661834 10.1111/j.1744-9987.2007.00491.x

[4] Thompson SM , Vella A , Thompson GB , et al. Selective arterial calcium stimulation with hepatic venous sampling differentiates insulinoma from nesidioblastosis. J Clin Endocrinol Metab. 2015;100:4189‐4197. 26312578 10.1210/jc.2015-2404 PMC4702445

[5] Witteles RM , Straus FH , Sugg SL , et al. Adult‐onset nesidioblastosis causing hypoglycemia. Arch Surg. 2001;136:656‐663. 11387003 10.1001/archsurg.136.6.656

[6] Arao T , Okada Y , Hirose A , Tanaka Y . A rare case of adult‐onset nesidioblastosis treated successfully with diazoxide. Endocr J. 2006;53:95‐100. 16543678 10.1507/endocrj.53.95

[7] Nadkarni M , Berns JS , Rudnick MR , Cohne RM . Hypoglycemia with hyperinsulinemia in a chronic hemodialysis patient following parathyroidectomy. Nephron. 1992;60:100‐103. 1738397 10.1159/000186712

[8] Shaer AJ . Management of hyperinsulinemia with diazoxide in an elderly hemodialysis patient. Nephron. 2001;89:337‐339. 11598399 10.1159/000046095

[9] Yorifuji T , Horikawa R , Hasegawa T , et al. Clinical practice guidelines for congenital hyperinsulinism. Clin Pediatr Endocrinol. 2017;26:127‐152. 28804205 10.1297/cpe.26.127 PMC5537210

[10] Shimizu M , Suzuki K , Tsuchida K , et al. Insulinoma in a patient with chronic renal failure due to type 2 diabetes mellitus treated effectively with diazoxide. Intern Med. 2015;54:621‐625. 25786453 10.2169/internalmedicine.54.3621

[11] Lamberts SWJ , van der Lely A‐J , de Herder WW , Hofland LJ . Octreotide. N Engl J Med. 1996;334:246‐254. 8532003 10.1056/NEJM199601253340408

[12] Yorifuji T , Kawakita R , Hosokawa Y , et al. Efficacy and safety of long‐term, continuous subcutaneous octreotide infusion for patients with different subtypes of K ATP‐channel hyperinsulinism. Clin Endocrinol. 2012;78:891‐897. 10.1111/cen.12075 23067144

[13] Kondo T , Tomita S , Adachi H , et al. A case of hyperinsulinemia of undetermined origin, successfully treated with long‐acting octreotide. Endocr J. 2005;52:511‐517. 16284426 10.1507/endocrj.52.511

